# Quality of labor and birth care in Sindh Province, Pakistan: Findings from direct observations at health facilities

**DOI:** 10.1371/journal.pone.0223701

**Published:** 2019-10-17

**Authors:** Sohail Agha, Laura Fitzgerald, Aslam Fareed, Presha Rajbhandari, Shaista Rahim, Farhana Shahid, Emma Williams, Wajiha Javed, Sheena Currie

**Affiliations:** 1 The Bill and Melinda Gates Foundation, Seattle, Washington, United States of America; 2 Jhpiego, Baltimore, Maryland, United States of America; 3 The Indus Hospital, Karachi, Pakistan; 4 Murshid Hospital, Karachi, Pakistan; 5 Jhpiego, Karachi, Pakistan; Public Health Foundation of India, INDIA

## Abstract

This study presents data from the first observation of labor, childbirth and immediate newborn care in a clinical setting in Sindh, the second most populous province of Pakistan. Trained midwives observed 310 births at 126 district level referral facilities and primary health care facilities in 10 districts of Sindh where the USAID-funded Maternal Child Health Integrated Program (MCHIP) was implemented. The facility participation rate was 78%. The findings show that monitoring vital signs during the initial examination was conducted for less than one-in-ten women. Infection prevention practices were only observed for one-in-four women. Modesty was preserved for less than half of women. In spite of an absence of monitoring during the first and second stages of labor, providers augmented labor with oxytocin in two-thirds of births. To prevent post-partum hemorrhage, oxytocin was administered within a minute of birth in 51% of cases. Immediate drying of the baby was nearly universal and eight out of ten babies were wrapped in a dry towel. Newborn vital signs and the baby’s weight were taken in one-in-ten cases. Breastfeeding was initiated during the first hour of birth in 18% of cases. A support-person was present during labor and birth for 90% of women. While quality of care is poor across all facilities, the provision of care at district-level referral facilities was even lower quality than at primary health care facilities. This is because *dais* or assistants without formal training provided labor, birth, and newborn care for 40% of deliveries during night shifts at referral facilities. This study found many examples of suboptimal practice by skilled birth attendants across all levels of health facilities. There remains an urgent need to improve quality of service provision among skilled birth attendants in Pakistan.

## Introduction

Over the last 20 plus years, substantial advances have been made in the reduction of maternal and newborn deaths worldwide [1], and during the same time many countries have measured increased coverage of facility-based care at the time of birth [2]. However, in many countries, increased access to institutional care has not resulted in commensurate reductions in mortality [3,4].

Access to institutional birth is insufficient if the quality of care is not provided at acceptable standards. Evidence suggests that improving the quality of care could dramatically reduce the numbers of maternal deaths, stillbirths, and neonatal deaths [4,5]. The quality of healthcare services is both a supply and demand issue, since community perceptions about poor quality healthcare are a well-known barrier to facility-based care [6,7].

In 2016, the WHO released Standards for Improving Quality of Maternal and Newborn Care in Health Facilities, which supported a framework for the quality of MNH care, and offered a conceptual model for considering and measuring the quality of MNH care [2]. The need for such a framework stems from substantial evidence in favor of focusing investments on the delivery of high-quality care in the intrapartum and immediate postpartum periods. Currently, available data on the quality of MNH care in developing countries relies largely on provider reports of their knowledge of various recommended practices or simulations of care provision in controlled settings [8,9]. Data based on observations of care provided in actual clinical settings is rare [10,11].

Within South Asia, the 2012–13 Demographic and Health Survey shows that Pakistan bears one of the highest burdens of both maternal mortality (276 deaths per 100,000 live births), and neonatal mortality (55 per 1,000 live births) [12]. One 2005 study in Pakistan described an increasing reliance of women on private sector facilities over public sector facilities, because of the widespread perception that the private sector provides better quality of care [13]. The few studies that have examined quality of care issues in Pakistan have focused on structural aspects of quality, including the availability of medicines, supplies and trained personnel and have tested providers on their knowledge of the standards of care but have not carefully examined provider performance in clinical settings [14].

To improve the quality of MNH care in an appropriate, context-specific manner, a detailed assessment of the actual situation in facilities is essential. To this end, USAID’s flagship maternal child health program, the Maternal Child Health Integrated Program (MCHIP) implemented an observation-based Quality of Care (QoC) survey in Sindh Province, Pakistan. The MCHIP program was operational in Pakistan from February 2013 to March 2018 [15]. This survey was designed to better characterize the process of service provision for antenatal, intrapartum, and postpartum care by facility-based providers. It is part of a small but growing literature which collects data on direct observation of the process of routine care provision for antenatal, labor and birth, and postnatal care in a low-income Asian country [16,17,18].

The purpose of this paper is to begin to fill gaps in the literature by providing detailed information on the provision of MNH care in Sindh, Pakistan as directly observed during service delivery. We focus here on provision of labor, birth, and immediate newborn care.

## Materials and methods

This cross-sectional observational study was conducted in 10 selected districts of Sindh (Dadu, Thatta, Khairpur, Tando Allah Yar, Tharkpar, Umerkot, Jacobabad, Sanghar, Naushero Feroz and Sukkur), the second most populous province of Pakistan [12], to establish baseline levels of quality of care prior to the implementation of a maternal and child health project supported by the United States Agency for International Development. The criteria for the selection of these districts were: strength of potential sub-grantee, limited availability of health services, lack of female staff at existing health facilities and the presence of other USAID partners in the district. These 10 districts were selected from a total of 29 districts in Sindh province.

A two-stage sampling strategy was used. In the first stage, between March and April 2014, a complete listing of all public and private health facilities providing maternal and child health services in these 10 districts was conducted. Facilities comprised of five types based on their management: 1) Department of Health-affiliated facilities at the primary and tertiary level; 2) government basic health units managed by a private organization called the People’s Primary Healthcare Initiative (PPHI); 3) private facilities led by community midwives (CMWs), who received an 18-month training to attend normal births and refer complications; 4) commercial for-profit clinics; and 5) non-governmental organization funded and managed facilities. Eighty cities and 2000 villages were visited by 66 study enumerators during the listing exercise. Enumerators used a brief structured instrument to collect information on facility hours of operation, facility type, services offered by the facility, and birth volume. Since the study utilized clinical observation of actual care provision during labor and birth, the birth volume was a key indicator for selecting facilities for observation. Due to resource constraints, it was not possible to station observers at each facility for more than 2 days. During the mapping exercise, data was collected on reported births in the last 24 hours, in the last 48 hours and in the last month. Mapping data was used to maximize the chances of observing at least one live birth at each facility during the 2-day observation period, taking into account the possibility of refusal from the facility director, provider or client.

### Instrument for clinical observation

In 2010–2011, MCHIP developed a routine labor and delivery clinical observation checklist, based on a previous multi-country study [19] and implemented this checklist across 7 countries in Africa [11]. It was further adapted for the Pakistan context to include recent evidence-based recommendations aligned with the Ministry of Health’s standard protocols. Observers used the checklist to collect data on many aspects of routine care in labor and delivery, including correct use of the partograph, active management of third stage of labor, infection prevention practices, and immediate essential newborn care. An observation ended when a woman was transferred to the postpartum recovery ward or discharged from the hospital—which sometimes occurred within a few hours of delivery.

### Observer training

A team of 23 female midwives or nurses with experience in intrapartum care received a 12-day training to prepare them to observe clinical services in an objective and standardized way. Observer training included a three-day workshop to refresh and update participants’ maternal and neonatal health knowledge and clinical skills specific to the intrapartum and postpartum period through interactive presentations, skills demonstrations and practice, and role plays. The subsequent four days focused on familiarizing observers with the study tools, allowing them practice with the use of study tools in simulated settings, and achieving inter-observer standardization. As part of the training, participants completed two days of direct observations of labor and birth at a high-volume teaching hospital. A final day was spent on protocols to be observed during fieldwork.

### Data collection

In the second stage, from April to June 2014, data collectors with clinical training observed births at 126 facilities. All facilities with at least two deliveries per day were contacted to seek permission for their participation in the study. The study aimed to observe labor and birth management for 300 births. Investigators made the most conservative sample size determination—since the study was to be applied across various indicators whose baseline levels were unknown—assuming baseline values of key indicators to be 50%. This would enable measurement of a minimal change of 12 percentage points with 80% power and 95% precision if the survey were repeated at the end of the project. In total, 310 deliveries took place at the selected health facilities during the time that trained observers were present. However, all stages of the delivery process could not be observed for all 310 women because some women reached the facility when the delivery process had already begun. As a result, the first stage of labor could only be observed for 248 women. A few women left the facility immediately after delivery and before the completion of other check-ups. This meant that immediate newborn care could only be observed for 300 women.

Two observers observed each labor and birth. In the case of events such as a new born being taken for resuscitation to another area, one of the two observers followed the newborn and observed care provision. Observers were provided guidance on observing the different stages of labor. The first stage of labor is considered to begin when the cervix is dilated at least 4 centimeters and the woman has at least 3 contractions in 10 minutes, with each contraction lasting at least 40 seconds. The second stage of labor starts when the cervix is fully dilated, at 10 cm, and ends with the birth of the baby.

Data were collected on paper forms. SPSS version 18 was used for data entry, and double entry of data was done to check the quality of data [20].

### Statistical analysis

Frequency distributions on indicators measuring the stages of the labor and birth process are presented, from the initial examination on admission in labor through the second and third stage of labor and extending to care of both women and newborns in the immediate postpartum period. For each stage, a summary score was created comprising a simple count of the number of elements of care observed. Analysis of variance was conducted to examine differences in mean scores by type of facility, type of provider, and time of shift (morning, evening or night) during which the observation took place. Stata version 14 was used for the data analysis [21]. ANOVA was conducted followed by pairwise comparisons of means. The latter is available as a postestimation command in Stata. For each test, a comparison of means was conducted using the category with the lowest score as the reference category. *P*-values are adjusted for multiple comparisons using Tukey’s HSD.

### Research ethics

The study research plan was reviewed and approved by the Johns Hopkins School of Public Health IRB (number 5524) and the Health Services Academy IRB in Pakistan. Written permission to collect data at the facility was asked of the facility director. Verbal informed consent was obtained from participants in this assessment–health care workers and patients whose care was observed. The literacy level among women observed at facilities was very low and a large proportion of those observed could not read and write. This was a major reason for taking verbal informed consent from women. Data collectors confirmed that consent was provided by both the health care providers and clients by ticking boxes on the data collection instrument. The IRBs approved verbal consent since a) this was an observational, minimal risk study, and b) low literacy was likely among the women to be observed.

## Results

### Site selection and participation (phase 1)

In total, 1631 facilities were listed during the mapping process and 1237 facilities reported that they offered labor and birth services. Of these 1237, 512 (41%) were public sector facilities, including referral facilities such as district headquarter hospitals (DHQ) and *tehsil* headquarter hospitals (THQ), and primary health care facilities, such as rural health centers and basic health units. 725 (59%) of the facilities offering labor and birth services comprised of private maternity homes, private clinics and private hospitals. The highest birth volume was at district headquarters hospitals (mean monthly births of 133), followed by tehsil headquarters hospitals (mean monthly births of 48) and private hospitals (mean monthly births of 25). Rural health centers averaged 13 births per month, basic health units averaged 4 births per month, private maternity homes and clinics averaged 13 births per month.

In total, 162 facilities with at least two deliveries per day were contacted and invited to participate in the study. Of all facilities contacted, 36 refused to participate, giving a 22% refusal rate. The subset of facilities which participated in the study but did not yield any births (n = 79) were broadly similar to the full set of facilities which participated (n = 126). Characteristics of facilities that refused to participate in the study, facilities that participated and facilities that participated but where no births were observed are shown in Table 1. Out of the 36 facilities that refused to participate, 30 (83%) were private facilities. By comparison, out of 126 facilities that participated in the study, 91 were private facilities (72%). Facilities that refused to participate were less likely to have Lady Health Workers attached to them (14% versus 31%) and less likely to offer inpatient services (47% versus 78%).

**Table 1:** Characteristics of facilities that refused to participate in the study, facilities that participated in the study and facilities that participated but did not yield any birth during the 2-day observation period.

	Facility participation status			Participated in study but no births observed (N = 79)
	Refused (N = 36)	Participated (N = 126)	Chi-square test p-value	
**Facility Type**				
Private clinic/hospital	30 (83.3%)	91 (72.2%)	Not significant	58 (73.4%)
Public clinic/hospital	6 (16.7%)	35 (27.8%)		21 (26.6%)
**Number of providers at facility**				
Between 1–3 providers	25 (69.4%)	78 (61.9%)	Not significant	51 (64.6%)
More than 3 providers	11 (30.6%)	48 (38.1%)		
**Lady health worker attached**				
Yes	5 (13.9%)	39 (30.9%)	p < 0.05	11 (13.9%)
No	31 (86.1%)	87 (69.1%)		68 (86.1%)
**24/7 outpatient services offered**				
Yes	26 (72.2%)	92 (73.0%)	Not significant	67 (84.8%)
No	10 (27.8%)	34 (27.0%)		12 (15.2%)
**24/7 inpatient services offered**				
Yes	17 (47.2%)	98 (77.8%)	p < 0.001	61 (77.2%)
No	19 (52.8%)	28 (22.2%)		18 (22.8%)

Facilities that participated in the study but where no births were observed (N = 79) are a subset of those who participated in the study (N = 126).

The refusal rate was higher in private facilities than in public facilities with 30 out of 121 private facilities refusing to participate (25% refusal rate) compared to 6 out of 41 public facilities (15% refusal rate).

### Observation of births (phase 2)

A total of 126 facilities were observed for 2 days each. As mentioned earlier, no births occurred at 79 facilities during the two days that trained observers were stationed there. In 47 of the 126 facilities (20 public and 27 private facilities), at least one birth was observed during the two-day period of observation. In total, 310 observations of labor and birth care were conducted at these 47 facilities. Overall, 137 births (44%) occurred during the morning shift, 64 (21%) during the evening shift and 109 (35%) during the evening shift.

**Fig 1:**
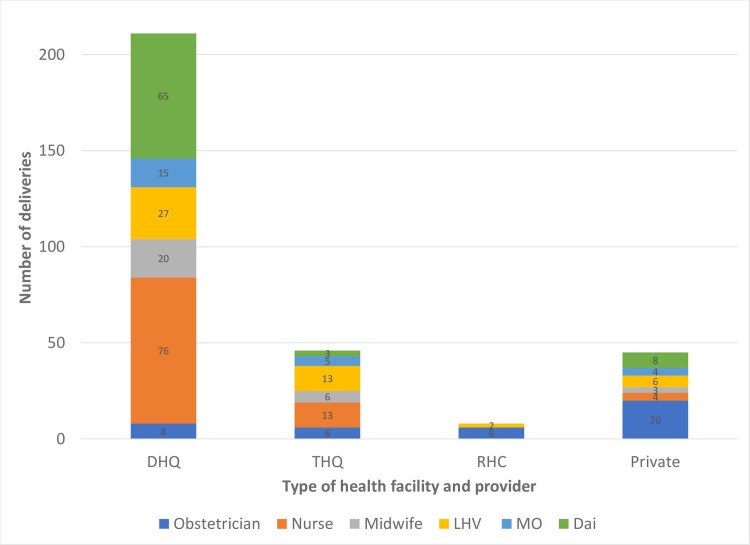
Number of deliveries observed at health facilities by type of primary health care provider.

Fig 1 shows the number of deliveries observed at health facility by type of primary health care provider. Of the 211 deliveries observed at DHQs, 65 (31%) were conducted by *dais* or assistants without formal training. By contrast, of the 45 deliveries observed at private facilities, 8 (18%) were conducted by *dais* or assistants without formal training. At private facilities, the highest proportion of deliveries conducted, 20 (44%), were by obstetricians.

**Fig 2:**
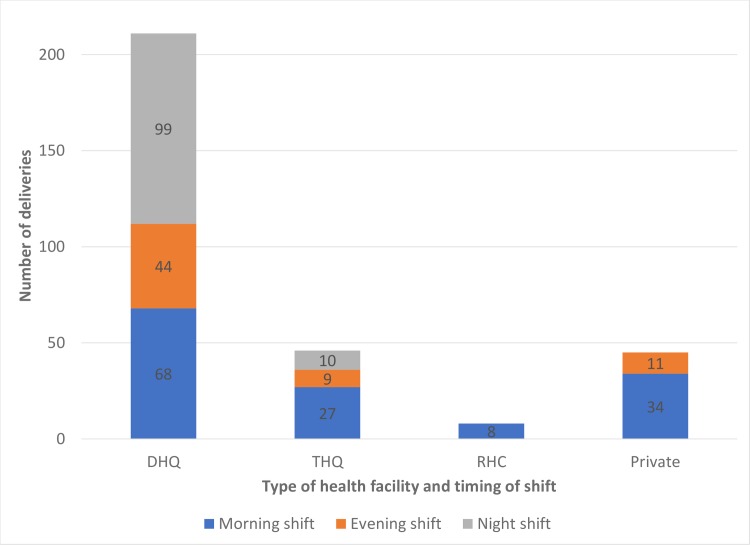
Number of deliveries observed at health facilities by timing of shift.

Fig 2 shows the number of deliveries conducted at health facility by timing of shift. Of the 211 deliveries observed at DHQs, 99 (47%) occurred during the night shift. By contrast, out of the 45 deliveries that were observed at private facilities, no deliveries were conducted during the night shift. At private facilities, 76% of deliveries occurred during the morning shift.

Our findings show that obstetricians provide a high proportion of labor and birth care in private facilities (44%), while *dais* or untrained assistants provide a substantial proportion of care at DHQs (31%). This is one of the primary reasons for better quality of care at private facilities compared with public facilities.

Table 2 shows indicators reflecting care provided to women in labor upon initial examination and during ongoing management of the first stage of labor. Basic infection prevention practices, such as hand washing prior to an initial examination (26%) or prior to a vaginal examination (27%) were practiced in a minority of births. However, the majority of providers wore sterile gloves to perform a vaginal examination (63%).

**Table 2:** Coverage of specific care practices during initial examination and during first stage labor.

	**First stage of labor: initial examination**	**N = 248**
1	Provider washes hands with soap and water or uses alcohol rub before any initial exam	64 (25.8%)
2	Explains procedures to woman (and support person if present) before proceeding	76 (30.6%)
3	Takes temperature on admission	29 (11.7%)
4	Takes pulse	30 (12.1%)
5	Takes blood pressure	98 (39.5%)
6	Performs general examination (e.g. for anemia)	78 (31.5%)
7	Checks fundal height with measuring tape	46 (18.5%)
8	Checks fetal presentation by palpation of abdomen	123 (49.6%)
9	Checks fetal heart rate with fetoscope/doppler/ultrasound	99 (39.9%)
10	Wash her/his hands with soap and water	68 (27.4%)
11	Wears sterile gloves for vaginal examination	156 (62.9%)
12	Informs the woman before conducting vaginal examination with respect	134 (54.0%)
13	Performs vaginal examination	230 (92.7%)
14	Informs pregnant woman of findings	121 (48.8%)
	Average score (out of 14)	5.5
	**Ongoing management of labor**	**N = 293**
1	At least once, provider explains what will happen in labor to woman/support person	105 (35.8%)
2	At least once, provider encourages woman to consume fluids/food during labor	116 (39.6%)
3	At least once, encourages woman to ambulate, assume different positions during labor	118 (40.3%)
4	Support person is present at some point during labor	265 (90.4%)
5	Partograph is used to monitor labor	9 (3.1%)
6	Provider gave at least one update on status and progress of labor	151 (51.5%)
7	Washes hands with soap and water or uses alcohol hand rub prior to any examination	84 (28.7%)
8	Uses curtains/ visual barriers to protect woman during exams, births, and procedures	119 (40.6%)
9	Wears sterile surgical gloves	148 (50.5%)
10	Drapes woman (one drape under buttocks, one over abdomen)	146 (49.8%)
11	Explains procedures to woman (support person) before proceeding	84 (28.7%)
12	Augments labor with oxytocin*	193 (67.2%)
13	Prepares uterotonic drug to use for AMTSL	261 (89.1%)
	Average score (out of 12)*	5.5

*Augmentation of labor not included in score

Vaginal examinations were performed for most women (93%), but only half the women were informed by the provider before the provider conducted a vaginal examination (54%), and about half were informed of the findings from the examination (49%). About 40% of women were encouraged to consume fluids during labor and to assume different positions during labor. About half of women were updated on the progress of labor (52%). Privacy was maintained (by using curtains or visual barriers) for 41% of women. Modesty was preserved for about half the women (by using a drape under the buttocks and over the abdomen) (50%).

Blood pressure was measured for 40% of women, and temperature and pulse were taken for 12% of women. A general physical examination was conducted for 32% of women. Fundal height was measured for less than one-fifth of women. Palpation of the abdomen to check fetal presentation and position was conducted in half the cases. The fetal heart rate was checked in 40% of cases. Only 3% of births included use of the partograph to monitor the progress of labor. Despite the absence of observed indications for labor augmentation, providers administered IV oxytocin to two thirds of women in labor (67%).

Table 3 shows the quality of care provided to women during the second and third stages of labor. Less than one-third of providers prepared for births by putting on protective clothing (31%). As observed during the first stage of labor, the rate of hand washing was 27%, while the use of sterile gloves was 53%.

**Table 3:** Coverage of specific care practices during second and third stages of labor and for the newborn after birth.

	**Second and third stages of labor**	**N = 305**
1	Provider puts on clean protective clothing in preparation for birth (goggles or gown)	94 (30.8%)
2	Washes hands with soap and water or uses alcohol hand rub before any examination	82 (26.9%)
3	Wears sterile surgical gloves	161 (52.8%)
4	Checks for another baby prior to giving the uterotonic	157 (51.5%)
5	Administers uterotonic	287 (94.1%)
6	Uterotonic administered within one minute of delivery of baby	154 (50.5%)
7	Checks uterine tone immediately following the delivery of the placenta	142 (46.6%)
8	Assesses completeness of the placenta and membranes	129 (42.3%)
9	Assesses for perineal and vaginal lacerations	196 (64.3%)
10	More than one health worker assisted with the birth	238 (78.0%)
11	Support person (companion) for mother present at birth	278 (91.1%)
	Average score (out of 11)	6.3
	**Immediate newborn care and health check**	**N = 300**
1	Immediately dries baby with towel	262 (87.3%)
2	Places baby on mother’s abdomen “skin to skin”	75 (25.0%)
3	Covers/wraps baby with dry towel	228 (76.0%)
4	Ties or clamps cord when pulsations stop, or by 2–3 minutes after birth	179 (59.7%)
5	Cuts cord with sterile blade or sterile scissors	166 (55.3%)
6	Applies 7.1% chlorhexidine digluconate gel to the cord stump	19 (6.3%)
7	Support person (companion) for mother present	245 (81.7%)
8	Mother informed of sex of baby	211 (70.3%)
9	Checks baby's temperature, by touch, 15 minutes after birth	40 (13.3%)
10	Checks baby's skin color 15 minutes after birth	60 (20.0%)
11	Takes mother's vital signs 15 minutes after birth	79 (26.3%)
12	Palpates uterus 15 minutes after delivery of placenta	100 (33.3%)
13	Baby kept skin to skin with mother for the first hour after birth	39 (13.0%)
14	Mother and newborn kept in same room after delivery (rooming-in)	203 (67.7%)
15	Baby not bathed in the first hour after delivery	247 (82.3%)
16	Breastfeeding initiated within the first hour after birth	53 (17.7%)
17	Weighs baby and documents the weight	40 (13.3%)
	Average score (out of 17)	7.5

For a majority of women in their second stage of labor, more than one health worker assisted with the birth (78%). In most cases a support person remained with the woman during this stage (91%).

Uterotonic medications were given to 94% of women for the prevention of postpartum hemorrhage during the third stage of labor. However, a uterotonic was administered during the first minute after birth in about half the cases. The provider checked the uterine tone following the delivery of the placenta in just under half of deliveries (47%) and the completeness of the placenta and membranes (to assess for retained parts or fragments) in 42% of cases.

Table 3 also shows the provision of newborn care within the first hour of birth. While drying the baby after birth to prevent hypothermia was practiced in 87% of births, skin-to-skin placement of the newborn on the mother’s abdomen was practiced in 25% of cases. For 82% of births, the baby was not bathed within the first hour of birth. Delayed cord clamping was practiced in 60% of births. The umbilical cord was cut with a sterile instrument in 55% of births. The newborn’s temperature was checked in 13% of cases, and 13% of babies were weighed during the first hour of birth. Breastfeeding was initiated during the first hour in 18% of cases. During the immediate postpartum period, the mother’s vital signs were taken in 26% of cases.

Table 4 shows quality of care summary scores during the multiple stages of care provided: initial examination during the first stage, ongoing management during the first stage, care provision during the second and third stage of labor, and care provision to both mother and newborn during the immediate postpartum period.

**Table 4:** Mean quality score by type of facility, type of provider, time of day during initial examination, stages of labor and immediate newborn care.

	Initial examination (n = 248)	Ongoing management (n = 293)	Second and third stage (n = 305)	Immediate newborn care (n = 300)
**Type of facility**				
District headquarter (reference)	4.6	5.0	6.1	7.0
Tehsil headquarter hospital	7.8***	7.3***	7.3**	9.8***
RHC/BHU	6.0	5.4	6.6	8.6
Private facility	6.8**	5.5	6.1	7.2
**Type of provider**				
Obstetrician	7.3***	6.2***	6.7***	8.3***
Staff nurse	5.5**	5.6**	6.4***	7.1*
Community midwife (CMW)	6.5**	5.8**	6.9***	8.9***
Lady health visitor (LHV)	6.4**	6.7***	7.8***	9.0***
Medical officer	5.1	5.4	6.8	8.2***
Dai/assistant (reference)	3.3	4.0	4.7	5.7
**Shift**				
Morning	6.3***	6.2***	6.7**	7.9*
Evening	5.9**	5.3	6.3	7.7
Night (reference)	3.9	4.7	5.8	6.8
**Total Score**	5.5	5.5	6.3	7.5

^*^p < 0.05

^**^p < 0.01

^***^p < 0.001

The quality of care score for the initial examination had a mean of 5.5 out of a possible maximum score 14. This score varied by type of facility, with THQs (7.8) and private hospitals (6.8) providing higher quality compared to DHQs (4.6), the reference category. The quality score varied by type of provider, with higher quality of care being provided by obstetricians (7.3), staff nurses (5.5), community midwives (6.5), LHVs (6.4) than by a *dai* or an assistant with no formal training (3.3), the reference category. The mean quality score varied by the time of day, with births in the morning shift receiving higher quality of care (6.3) than births at night (3.9), the reference category. The lower quality of care during the night shift is probably due to provision of care by *dais* during the night shift.

The pattern was similar for quality of care provided during the ongoing management of labor, the second and third stage of labor and immediate newborn care with quality of care being higher at THQs than at DHQs, the reference category. The quality of care provided by obstetricians, staff nurses, CMWs and LHVs was higher than what was provided by *dais* or untrained assistants, the reference category. The quality of care provided was higher in the morning shift than in the night shift, the reference category.

## Discussion

These findings provide important insights regarding the quality of care provided during routine intrapartum and immediate postpartum period (defined here as within the first hour following delivery). Findings across the continuum of care are discussed according to three major themes: infection prevention and control practices, routine assessment and monitoring, and the provision of care that is respectful and in accordance with recommended standards.

### First stage of labor: Initial examination

During the initial examination in the first stage of labor, our findings show large gaps in provider practices related to infection prevention such as hand washing before and after contact with patients and appropriate use of sterile gloves. Taking measures to prevent infection from the first point of contact with clients has implications for longer term outcomes, especially considering that sepsis is the second leading cause of both maternal and newborn mortality in Pakistan and responsible for 14% of maternal deaths and 20% of neonatal deaths [22]. A 2009 review found that improved hand hygiene alone could reduce facility-acquired infections by up to 40% [23]. Low-cost investments in infection prevention–such as the introduction of infection prevention guidelines and facility-based infection control teams–could dramatically improve outcomes for women and newborns in Pakistan [24].

Completing the recommended steps of a routine maternal and fetal assessment during the initial examination are also essential in order to establish the overall health status of the woman and fetus, identify any potential complications, and to decide about the kind of care a woman and her baby will need. A number of gaps were observed in relation to routine assessment during this period. For instance, only 12% of providers in our study checked the mother’s temperature on initial admission, suggesting that in addition to not taking steps to prevent infection, many providers are also not screening for early signs of infection. Consistent provider screening of women’s blood pressure to assess for hypertension was observed in only 40% of laboring women during the initial examination. This presents a grave missed opportunity to assess for pre-eclampsia and eclampsia, the third leading cause of maternal death in Pakistan, which accounts for 10% of maternal mortality [22].

Select measures of Respectful Maternity Care (RMC) and provision of recommended care practices during the initial examination, and throughout the intra and postpartum periods, also revealed room for improvement. Our study provides a unique opportunity to reflect on several specific measures of RMC including whether providers greet clients, ensure privacy, ask permission before invasive procedures and provide information about findings. Increasing global attention to RMC sheds light on the critical importance of measures of quality that capture attitudinal dimensions of care [25,26]. Given the sensitivity surrounding the issue, and the relatively recent focus on its essential link to quality of care, limited global data about RMC has been published.

### Ongoing management of labor

Overall, findings during the ongoing labor management were similar to those observed during the initial examination, although some measures of RMC during this period were quite high. Ninety percent of women were accompanied by support people of their choice, an indicator of RMC known to decrease the length of labor and improve outcomes [27]. However, privacy measures such as draping and use of visual barriers were not standard. Rates of routine ongoing monitoring during labor were also low. Since labor is not routinely monitored and documented in Sindh, prolonged and obstructed labors may not be consistently diagnosed–leading to missed opportunities for early, coordinated referrals and timely links to advanced care [24].

Perhaps the most significant, and concerning, study finding during observation of ongoing labor management was the frequency with which labor was augmented with oxytocin. Augmentation, which should only be practiced only when it is medically indicated to strengthen contractions [28], was practiced in 67% of births. If labor augmentation is being performed in two-thirds of cases without routine labor monitoring, one can presume that providers are attempting to speed the progress of labor without ensuring the safety of the woman and fetus [29]. Further, labor should be augmented only after a thorough examination of the woman and fetus, and only performed in those facilities where surgical intervention is readily available should a complication arise. As noted by WHO (2014), while interventions within the context of augmentation of labor may be beneficial, their inappropriate use can cause harm [28].

### Second and third stages of labor

Infection prevention practices during the second and third stage of labor continued to be low—as evidenced by underuse of protective gown and gloves and sterile equipment to conduct deliveries and to cut the umbilical cord. Routine monitoring of maternal and fetal/newborn wellbeing throughout the second and third stages of labor, essential to identifying and responding to complications, is largely not taking place in our sample health facilities in. Pakistan has the third highest burden of total number of stillbirths [30] and one of the highest stillbirth rates [31]. The stillbirth rate is an indicator which provides a proxy measure of the quality of intrapartum monitoring and care. Birth asphyxia, a leading cause of neonatal mortality in Pakistan, could be reduced through improved intrapartum monitoring. Improving the uptake of labor monitoring presents an immediate opportunity to strengthen clinical decision-making for women and newborns [5].

However, some essential preventative care measures are being consistently performed during the intrapartum period. Uterotonic use is the most significant component of active management of the third stage of labor and helps prevent postpartum hemorrhage [19], the leading cause of maternal death in Pakistan [22]. Our study finds that uterotonic use is nearly universal in facility births in Sindh province (94%), although it is given within the first minute of the baby’s birth—as recommended—in only half the cases.

### Immediate newborn care and health check

Our study found that routine monitoring and assessment continues to be an issue during the first hour after birth. Providers rarely checked newborn vital signs: the newborn’s temperature was checked in 13% of cases; skin color was checked for only 20% of newborns. Performing routine assessments, including checking vital signs and conducting routine recommended physical examinations, allows providers to identify warning signs early and take prompt action to prevent complications. Educating mothers about danger signs for themselves and their newborns and assisting families to develop plans for seeking care in the event of a complication, can also improve outcomes for women and newborns.

Published in the Lancet Every Newborn Series 2014, global experts present a package of care around the time of birth that save the most lives [32]. Many of the included high-impact interventions require little or no additional supplies or equipment, and minimal provider training. For instance, increasing the use of thermal care measures such as placing newborns skin-to-skin immediately after birth have particular implications in Pakistan, the country with the world’s highest proportion of low birth weight babies (who struggle to maintain body temperature). Our study also found that only 13% of babies are weighed during the first hour after birth. Strengthening routine weighing of babies in the immediate postpartum period could improve the identification and early management of low birthweight babies and those with special needs. Immediate and exclusive breastfeeding, one of the most important interventions for ensuring newborn survival, was observed in less than one-fifth of cases (18%) in this study. Supporting the routine use of these simple, yet powerful, interventions requires little additional financial, material, or infrastructural investments.

### Human resources, learning and performance needs

This study found that about 40% of births during the night shift at DHQs are conducted by *dais* or assistants lacking formal training, even though this practice is not authorized by local policies–and this contributed to the lower quality of care observed at DHQs compared to THQs. This is consistent with literature that recognizes concerns about standardized, high quality 24-hour care in south Asia, with particular personnel shortages and higher levels of patient mistreatment documented at night [33–35]. While official staffing policies may be more evenly distributed across shifts, these findings suggest that skilled birth attendants may preferentially work during the day. Ensuring the availability of skilled birth attendants to care for women and newborns at night is an urgent and important action that will lead to an increase in the quality of clinical care for a significant proportion of women and babies accessing care in referral facilities in the public sector. Such a change could lead to an overall improvement in quality as DHQs are the most frequented facilities for labor and birth care in the ten rural districts of Sindh in which this study was conducted. Improvements in health workforce planning would also likely have an immediate and positive effect on the delivery of higher quality care. In particular, our study suggests that finding ways to increase the number of skilled birth attendants who care for women and newborns at night could improve the quality of care, and client health outcomes.

This study sheds light on the quality of care provision in the private sector in Sindh, something few surveys have been able to do to date. While the actual quality of clinical care in the public and private sectors is comparable, it is interesting that a much higher proportion of deliveries in the private sector compared with the public sector (44% versus 8%) are attended by obstetricians. This suggests that private facilities cater to a segment of the population which perceives care provided by obstetricians to be of better quality than care provided by nurses or midwives who attend the majority of births at public facilities. Given that no births were observed at private facilities at night, and that births were only observed in THQs and DHQs at night, it is possible that obstetricians working in private health facilities may only be conducting births which can be planned for daytime hours (i.e. C-sections). The latter may both provide better financial remuneration and convenience for the obstetrician. The lack of a statistically significant difference in the overall quality of clinical care provided in the public and private sectors in Sindh is not surprising given that many providers work in the public sector while simultaneously maintaining private practices [36].

This study found many examples of suboptimal practice by skilled birth attendants providing care during labor and birth, a time when utilization of evidence-based, high impact interventions is essential. This is consistent with assessments of competence of providers in Pakistan, as in other countries of South Asia, which show low levels of knowledge of basic obstetric and newborn care [9,14,37]. Challenges to provision of high-quality MNH care regionally extend well beyond lack of health worker knowledge and skills and include lack of essential supplies, equipment, and services, lack of clinical protocols, as well as challenging work environments [37–39]. Data from Afghanistan, Bangladesh, and India describe facility cultures marked with cronyism, nepotism, “fear and blame”, and bribery, as well as unreasonable workloads and overcrowding [37–39]. Significant gaps in pre-service education, particularly for community midwives, are well-documented [40–41]. And, a 2015 study in Karachi, Pakistan found that midwives suffered from low motivation due to low salaries, negative societal perceptions, and limited opportunities for higher education [42]. Addressing these infrastructural, workplace culture, and health workforce planning gaps is essential, and must happen alongside efforts to build in-service healthworker capacity, in order to achieve measurable improvements in client outcomes.

Although overall quality of care provided was low, it is notable that community midwives and lady health visitors provided quality of care comparable to obstetricians and medical officers, especially for the 2nd and 3rd stages of labor and for immediate newborn care. This is an extremely important finding, with implications for task shifting.

### Study strengths and limitations

Our study contributes to the literature by possibly being the first in Pakistan to observe actual provision of MNH care at the point of contact in a clinical setting. The observation started from the initial examination of the woman upon presentation, ongoing monitoring during the three stages of labor and until the period immediately after birth. The results provide estimates on adherence to globally accepted standards for clinical practice. Unlike previous QoC studies that focused on public sector factors, this study provides data on care provision in both the public and private sectors.

While the actual observation of care provision is thought to be a reliable method of documenting the process of care provision (especially in contexts where record-keeping is poor), it can also lead to biased measurement with providers performing better than usual because of being under observation–also referred to as the “Hawthorne Effect”. Thus, our study probably reflects the best-case scenario in terms of the quality of care provided at health facilities in Sindh, making the findings all the more striking. Recent evidence suggests that alternate methods for measuring the quality of care, such as the use of standardized (mock) patients, may yield a truer picture of routine care and avoid the Hawthorne Effect [43]. However, this method was not feasible for our study design, due to logistical, financial, and ethical constraints.

One limitation of this study is the higher refusal rate in private sector facilities (25%) compared to public sector facilities (15%). The private health sector is unregulated in Pakistan, and it is challenging to collect data from private maternal and child health facilities in Sindh.

## Conclusion

As in the case of several other developing countries, institutional deliveries have increased significantly in Pakistan without commensurate reductions in morbidity and mortality. Our study has helped develop a better understanding of the quality of services provided to mothers and newborns in Pakistan. Overall, the quality of care provided to mothers and newborns in both public and private sector health facilities in Sindh, the second most populous province of Pakistan, is extremely poor. The virtual absence of routine monitoring during labor and birth, as reflected by providers not taking vital signs of women and newborns, is troubling. By not identifying factors that may substantially increase the risk of maternal and newborn morbidity and mortality, providers are missing a tremendous opportunity for care provision. Quality of care is poorer in referral facilities than in primary health care facilities, due to untrained assistants or *dais* being left unsupervised to provide services during night shifts. Our findings also show that there is not much difference in the quality of care provided by obstetricians and community midwives or lady health visitors, although there appears to be a preference for obstetricians in private health care facilities.

The implications of our study are similar to those of a study in Uttar Pradesh, India [16]. There is a need to investigate reasons for untrained assistants, *dais*, being allowed to provide a substantial proportion of care in referral facilities, a need to recognize that there is a the widespread lack of adherence to recommended protocols in all types of health facilities, and to determine what types of tailored solutions combining supervision and accountability with provider behavior change are needed to rectify the situation.
